# *Plasmodium vivax* molecular diagnostics in community surveys: pitfalls and solutions

**DOI:** 10.1186/s12936-018-2201-0

**Published:** 2018-01-30

**Authors:** Maria Gruenberg, Clara Antunes Moniz, Natalie Ellen Hofmann, Rahel Wampfler, Cristian Koepfli, Ivo Mueller, Wuelton Marcelo Monteiro, Marcus Lacerda, Gisely Cardoso de Melo, Andrea Kuehn, Andre M. Siqueira, Ingrid Felger

**Affiliations:** 1 Swiss Tropical and Public Health Institute, Socinstrasse 57, 4002 Basel, Switzerland; 2 University of Basel, Basel, Switzerland; 3 Walter and Eliza Hall Institute of Medical Research, Parkville, VIC Australia; 4 Fundação de Medicina Tropical Dr. Heitor Vieira Dourado (FMT-HVD), Manaus, Brazil; 5 Universidade do Estado do Amazonas, Manaus, Brazil; 6 Instituto Nacional de Infectologia, Evandro Chagas, Fiocruz, Rio de Janeiro, Brazil

**Keywords:** *Plasmodium vivax*, Surveillance, Molecular diagnostics, Mitochondrial DNA, 18S rRNA transcripts, LAMP, Quantification, Gametocytes

## Abstract

A distinctive feature of *Plasmodium vivax* infections is the overall low parasite density in peripheral blood. Thus, identifying asymptomatic infected individuals in endemic communities requires diagnostic tests with high sensitivity. The detection limits of molecular diagnostic tests are primarily defined by the volume of blood analysed and by the copy number of the amplified molecular marker serving as the template for amplification. By using mitochondrial DNA as the multi-copy template, the detection limit can be improved more than tenfold, compared to standard 18S rRNA targets, thereby allowing detection of lower parasite densities. In a very low transmission area in Brazil, application of a mitochondrial DNA-based assay increased prevalence from 4.9 to 6.5%. The usefulness of molecular tests in malaria epidemiological studies is widely recognized, especially when precise prevalence rates are desired. Of concern, however, is the challenge of demonstrating test accuracy and quality control for samples with very low parasite densities. In this case, chance effects in template distribution around the detection limit constrain reproducibility. Rigorous assessment of false positive and false negative test results is, therefore, required to prevent over- or under-estimation of parasite prevalence in epidemiological studies or when monitoring interventions.

**Electronic supplementary material:**

The online version of this article (10.1186/s12936-018-2201-0) contains supplementary material, which is available to authorized users.

## Background

**Fig. 1:**
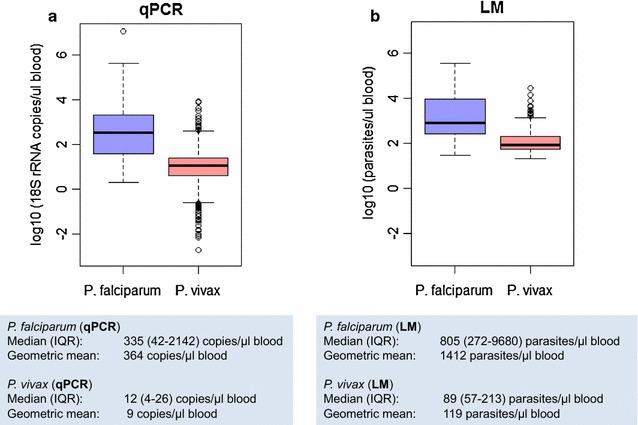
Parasite densities of *P. falciparum* and *P. vivax* measured by qPCR (**a**) and light microscopy (**b**) in community samples from PNG (5–9 years old children from 6 neighbouring villages) (Data taken from [41])

Parasite densities in *Plasmodium vivax* infections are generally lower compared to *Plasmodium falciparum* densities. For example, in Papua New Guinea (PNG), among children living in an area with similar *P. falciparum* and *P. vivax* transmission rates, the difference in mean parasite density between both species was ten-fold by light microscopy (LM) and 30-fold by quantitative PCR (qPCR) (Fig. 1) [1]. A similar difference in densities between both species was observed in the general population [2]. The lower parasite densities of *P. vivax* can be explained by the strict host cell preference of this species, which infects only reticulocytes that account for less than 1% of all erythrocytes. *P. falciparum* is less restricted in host cell selection and, thus, can reach higher densities. Moreover, age trends in infection prevalence and clinical incidence suggest an earlier acquisition of clinical immunity and more effective control of parasitaemia for *P. vivax* compared to *P. falciparum* [3]. In a cohort of young children from PNG (1–4 years), the incidence of clinical *P. vivax* episodes decreased significantly by the second year of life, whereas the incidence of clinical *P. falciparum* episodes continued to rise up to 4 years of age [3]. This indicates that young children in endemic areas acquire the ability to effectively control *P. vivax* parasitaemia early in life.

The overall lower density of *P. vivax* compared to *P. falciparum* plays a critical role in limiting the test sensitivity of diagnostic methods used to measure parasite prevalence, such as light microscopy (LM), rapid diagnostic test (RDT) and quantitative PCR (qPCR). A systematic review of sub-microscopic *P. vivax* infections showed that in cross-sectional surveys from diverse transmission settings, an average 67% of all *P. vivax* infections were sub-microscopic and would thus remain undetected by LM [4]. As for *P. falciparum*, a negative relationship was observed between the proportion of sub-microscopic infections and prevalence by LM. In view of the lower *P. vivax* densities overall, molecular-based diagnostic tools are even more relevant for detection of *P. vivax* than for *P. falciparum,* particularly in areas of low transmission. In this paper, diagnostics for detecting *P. vivax* blood stage infections are discussed. Hypnozoites, another hallmark of *P. vivax* infections, cannot be detected by current diagnostic methods.

## Diagnostic tools for surveillance

LM has traditionally been the gold standard for malaria epidemiology, while prevalence by LM has been used to describe malaria transmission levels globally. Having made substantial progress in malaria control, interventions have shifted focus from targeting clinical cases only towards identifying and treating asymptomatic parasite carriers, as well. Hence, the extent of sub-microscopic *Plasmodium* infections and the ability of molecular diagnostic tools to detect them have increasingly attracted attention [4, 5]. The limited sensitivity of LM compared to molecular diagnosis derives from the very small volume of blood (0.025–0.0625 µL whole blood) examined per blood slide for parasite counts in field studies [6]. Molecular techniques permit examination of an equivalent of 5–10 µL whole blood, which increases test sensitivity substantially. However, using increasingly high blood volumes in molecular diagnostic tests would not necessarily result in a linear increase in sensitivity, as large amounts of human genomic DNA will act as PCR inhibitor. Attempts to maximize molecular test sensitivity by increasing the input material to several mL of venous blood would require depletion of human white blood cells [7].

To increase the comparability of molecular-epidemiological data generated across different field sites and laboratories, a defined set of experimental details should be included in any report. These recommended specifications are presented in Box 1.

**Box 1:** Recommended reporting of experimental details in molecular-epidemiological studies

Requirement for publication	Experimental information to be reported
Imperative	*Sampling details of finger prick or venous blood sampling*; e.g. type of filter paper (treated or not), microtainer/tubes (heparin, EDTA), storage solution (RNAprotect/Trizol)
	*Description of extraction method*; e.g. extracted blood volume, spin columns, chelex, type of DNAse treatment of purified RNA
	*Resuspension/elution volume for extracted DNA or RNA*
	*Description of molecular target*; e.g. Gene ID, amplicon size, primer and probe sequences
	*Reagent concentrations and total reaction volume*; e.g. concentrations of primer/probe, template volume added to amplification reaction
	*Standard used for quantification*; e.g. parasite trendline (with stage composition), NIBSC WHO reference standard, plasmid (linearized/supercoiled)
	*Clear definition of quantification results*; e.g. method used for conversion of copy numbers into parasites/µL blood, clear denominator for “template copy number/µL whole blood or DNA”
	*Assay performance parameters*; e.g. specificity, assay LOD, PCR efficiency
Optional	*Storage conditions and time prior to extraction*, e.g. of filter papers or whole blood, temperature, desiccant, particularly for filter papers
	*Reproducibility*; e.g. results from duplicates or triplicates
	*Comparison to LM*; e.g. correlation of parasite counts/µL blood to copy numbers/µL blood

Technical details should be reported according to MIQE guidelines: minimum information for publication of quantitative real-time PCR experiments [50]

For diagnosing community samples, the desired profile of a diagnostic test differs from that of clinical management. For example, control interventions targeting all individuals who may contribute to malaria transmission require robust diagnosis of low density infections in asymptomatic parasite carriers. In response to this need, experts in *P. vivax* diagnostics and epidemiology recently defined target product profiles (TPP) for *P. vivax* diagnosis in malaria epidemiological field work [8]. Three distinct TPPs for the next generation of *P. vivax* diagnostic tests for control and elimination were generated under the leadership of the Foundation for Innovative Diagnostics (FIND). Each TPP addressed a particular diagnostic task: (i) a point-of-care tool for clinical case management (e.g. an ultra-sensitive RDT for *P. vivax*); (ii) a molecular ultra-sensitive test for mobile teams engaged in surveillance-response activities targeting asymptomatic carriers that can be performed rapidly, in a single tube and at the point-of-care; and (iii) a molecular ultra-sensitive test for large-scale surveillance activities or research where the time to result is not critical, and that can be performed at high throughput and low cost at a core facility [8]. Molecular assays that target multiple copies per genome have the potential to increase test sensitivity sufficiently to allow pooling of several samples without compromising test sensitivity. Using pooling for the last two tasks can reduce costs, particularly in areas of low *P. vivax* prevalence (< 2%).

## *Plasmodium vivax* 18S rRNA as marker gene for DNA- and RNA-based detection

18S rRNA genes are the standard molecular markers for differentiating *Plasmodium* species. In the two *P. vivax* reference genomes sequenced, Sal1 and P01, three distinct 18S rRNA copies exist and are expressed in different developmental stages (Additional file 1: Table S1) [9]. However, a widely used Pv18S RNA assay [10] targets only one of the three Pv18S rRNA copies by qPCR.

In contrast to *P. falciparum*, schizont stages of *P. vivax* are found in the peripheral blood [11]. As schizonts can contain 16–24 genomes, a direct conversion from copy number to parasite counts will not be accurate. This issue has been investigated using digital droplet PCR (ddPCR), a technology that permits absolute quantification of template DNA [12]. A very strong correlation (R = 0.86) was found for *P. vivax* quantification by the two molecular methods, ddPCR and standard Pv18S rRNA qPCR [12]. The correlation between *P. vivax* microscopy counts and quantification by ddPCR and qPCR was good (R = 0.72 and R = 0.73, P < 0.0001) [12]. Similar correlations were observed for *P. falciparum*, thus, it seems that the occasional presence of *P. vivax* late stages in finger-prick blood samples does not substantially affect molecular quantification. The number of Pv18S rRNA gene copies detected per parasite was determined by comparing with LM data. On average, one Pv18S rRNA copy per parasite was measured by ddPCR. As multiple genomes should be detected per schizont, a loss or damage of genomic copies during DNA extraction has to be assumed [12].

The same Pv18S rRNA assay can also be used to target Pv18S rRNA transcripts instead of the genes themselves [10, 13, 14]. Targeting RNA transcripts amplifies sensitivity, since each ribosome carries one copy of rRNA, which amounts to thousands of 18S rRNA transcripts per cell. For *P. falciparum,* a factor to convert Pf18S rRNA transcripts into parasite counts was established using synchronous cultured parasites [13]. 10^4^ 18S rRNA transcripts were measured per ring stage parasite; this number remained constant for the initial 24-h of the life cycle. As *P. vivax* cannot be readily cultured in vitro, a conversion factor for *P. vivax* only could be estimated using parasite counts by LM from field samples [10]. Correlation between microscopic *P. vivax* counts and the number of Pv18S rRNA transcripts was moderate (r^2^ = 0.44) [10]. The discrepancies between LM and molecular quantification might derive from between-sample variation in parasite stage composition or from RNA content per parasite. Additionally, variable conditions of RNA preservation and sample storage in the field affect the quality of extracted RNA, making RNA-based quantification less reliable compared to DNA-based quantification.

## Problems caused by targeting transcripts of *Plasmodium vivax* 18S rRNA

During nucleic acid extraction, there is an inherent risk of contaminating parasite negative samples handled alongside parasite positive samples. Cross-contamination can occur even without pipetting errors, by spreading aerosols when handling highly concentrated nucleic acids. Thus, the greatest care needs to be taken when working with clinical samples for both DNA and RNA template molecules. In view of the exceedingly high copy numbers of ribosomal RNA transcripts compared to genomic 18S rRNA copies, this contamination threat is potentiated by working at RNA level, leading to false positivity [10, 15].

**Fig. 2:**
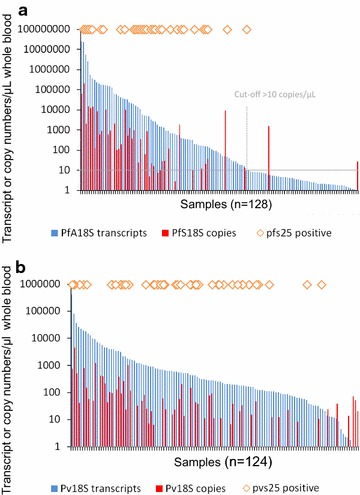
Detection of 18S rRNA genomic copies compared to 18S rRNA transcripts. Data used for this blot derived from earlier published work [10]. Dashed line: choice of cut-off (> 10 transcripts per reaction)

This risk of cross-contamination was addressed in a cross-sectional survey of 315 children from PNG, where DNA-based and RNA-based detection and quantification were compared for *P. vivax* and *P. falciparum* [10]. Figure 2 shows the number of *P. falciparum* and *P. vivax* 18S rRNA transcripts in study participants, plotted by decreasing number of 18S rRNA transcripts. For *P. falciparum*, transcript numbers trailed off over a substantial number of samples at the lower density end (< 10 transcripts, 40% of all positive samples). This was not observed for *P. vivax*, which may be explained by the lower median parasite density (8 times lower by LM and by qPCR) in *P. vivax* positive samples compared to *P. falciparum* infections of the same study.

The potential for cross-contamination between the wells of an RNA extraction plate was investigated by analysing large numbers of negative controls (phosphate-buffered saline, PBS) in parallel with interspersed wells containing high density *P. falciparum* 3D7 culture, mimicking high density clinical infections. False positive results were observed in some of the wells neighbouring high-density samples. These confirmed false positives were typically characterized by transcript numbers < 10 transcript copies/µL and in very few exceptions < 50 copies/µL. Such high parasitaemia, as used in these control experiments, might be reached only rarely in community samples, yet, this observation calls for great care during extraction and pipetting. Analysis of the PNG field samples (Fig. 2) led to the conclusion that for *P. falciparum,* a cut-off for positivity was required when detecting Pf18S rRNA transcripts, to exclude false positive results caused by low-key contamination from the few intermittent high density infections. Based on the distribution of Pv18S rRNA transcript copy numbers (Fig. 2b), a cut-off for *P. vivax* RNA-based parasite detection and quantification did not seem necessary. In summary, the pitfalls of RNA-based diagnosis do not reject parasite detection based on 18S rRNA transcripts, but rather call for awareness, utmost caution and well-controlled experimental procedures.

## *Plasmodium vivax* assays targeting multi-copy templates

High-copy genomic sequences can serve as new PCR targets for the detection of malaria infections, providing increased sensitivity over single- or low-copy 18S rRNA genes, without the pitfalls of RNA-based amplification. Furthermore, multi-copy markers have the potential to allow sample pooling without jeopardizing test sensitivity. This would be particularly favourable in the context of increasingly large study sizes required for community surveys conducted in elimination settings with low prevalence rates.

The *P. vivax* genome was mined to identify species-specific, repetitive sequences. The best target identified was the non-coding subtelomeric repeat sequence Pvr47, which occurs in 14 copies per *P. vivax* Sal1 genome [16]. A Pvr47-based single-step PCR assay was almost as sensitive as nested PCR targeting the *P. vivax* 18S rRNA when visualized in an agarose gel [16]. Attempts to use Pvr47 for designing a LAMP assay failed due to specificity problems [17]. When the Pvr47 assay was used to detect *P. vivax* in *Anopheles* spp. mosquitoes, non-specific bands and sequences were produced [18].

A number of attempts were made to identify other multi-copy markers for detection of *P. vivax*. Similar to a qPCR assay developed for ultra-sensitive detection of *P. falciparum* that targets the conserved C-terminus of the *var* gene family [19], *P. vivax* candidates were sought among the *vir/pir* multigene family [20, 21]. However, genetic diversity among members of this family is extremely large, such that no DNA stretches of sufficient sequence conservation and size for primer and probe design were identified [22]. Recently, a revised *P. vivax* reference genome (P01) with improved assembly of the subtelomeres became available [23]; new attempts are currently underway to identify multi-copy targets.

In view of the high genetic variability in repeated genomic regions and in the *vir* genes of *P. vivax*, mitochondrial DNA (mtDNA) offers relatively conserved regions for primer design, as well as sufficient diversity for distinguishing the different *Plasmodium* species. The mitochondrial genome of malaria parasites is present in multiple copies per cell, contained in a single mitochondrion. For *P. falciparum,* the total number per ring stage parasite is about 20 mitochondrial genomes [24]. The bulk of these copies are present in linear tandem arrays of 3–4 units [25]. Replication occurs simultaneously with the nuclear genome, about 24-h post invasion. For *P. falciparum* with sequestered late stages, the gain in sensitivity from using a mitochondrial marker compared to nuclear markers is potentially limited, as the multiple copies of mitochondrial DNA (mtDNA) are not distributed independently, but in six molecules, each composed of the 3–4 tandem repeat units of mtDNA. In *P. vivax*, however, late stages with multiple genomes and the replicating mitochondrial genomes are also present in peripheral blood. While the organization of *P. vivax* mtDNA is not known, a substantial template multiplication factor can be expected. Thus, the gain in sensitivity from targeting the mitochondrial genome might be greater for *P. vivax* than for *P. falciparum*.

A number of assays for diagnosing *P. vivax* have targeted mtDNA: one-step PCR; loop-mediated isothermal amplification (LAMP) or qPCR, targeting the cytochrome C oxidase I gene (*cox1*) [26–29]; genus-specific PCR, targeting non-coding regions between the cytochrome B gene (*cytB*) and *cox1* [30]; nested PCR, targeting cytochrome C oxidase III (*cox3*) [31]; and genus-specific nested PCR, targeting the *cyt B* gene, followed by sequencing of the PCR product or restriction fragment length polymorphism (PCR–RFLP) for species identification [32, 33].

## Targeting mitochondrial DNA by qPCR in cross-sectional samples from Brazil

**Fig. 3:**
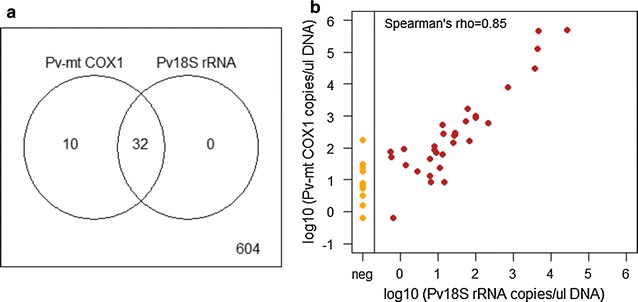
Comparison of Pv-mtCOX1 and Pv18S rRNA assays performed in parallel in 604 community samples from Brazil. **a** Overlap in positivity by Pv-mtCOX1 and Pv18S rRNA qPCR. **b** Correlation of log10 template copy numbers detected by Pv-mtCOX1 and Pv18S rRNA qPCR

A qPCR assay was designed to target the *P. vivax* mitochondrial *cox1* gene (Pv-mtCOX1 qPCR, Additional file 2: Table S2). This assay showed performance characteristics superior to Pv 18S rRNA qPCR (Additional file 3: Table S3, Additional file 4: Table S4). Some 604 samples collected from a cross-sectional survey in the Amazonas region, Brazil, in 2014 were re-analysed with Pv-mtCOX1 qPCR to investigate the effect of applying highly sensitive DNA-based parasite detection to community samples and asymptomatic parasite carriers. The number of *P. vivax* positive samples differed substantially by assay and 23.8% of positive samples were only detected by the Pv-mtCOX1 assay (Fig. 3a). Overall positivity was very low, with 4.9% (CI_95_ [3.4–6.9%]) of samples testing positive by 18S rRNA qPCR and 6.5% (CI_95_ [4.7–8.7%]) by Pv-mtCOX1 qPCR. In samples deemed positive by both assays, the correlation of template copy numbers obtained by the two assays was good (Spearman’s rho = 0.85, red data points in Fig. 3b).

**Fig. 4:**
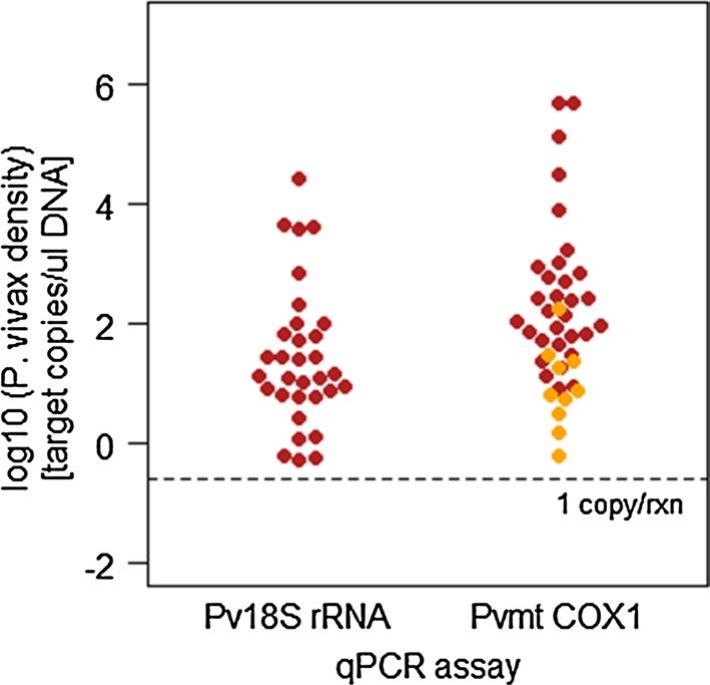
Copy numbers of each marker gene detected per sample. Each dot represents one sample, red indicates all samples positive for Pv18S rRNA qPCR, orange indicates samples detected only by Pv-mtCOX1 qPCR. Dashed line: Molecular assays have a theoretical LOD, i.e., at least 1 template copy must be present per PCR reaction

To investigate the relationship between template copy number and positivity, copy numbers were plotted for all positive samples for both assays (Fig. 4; Additional file 5: Figure S1). The median gene copy number for Pv-mtCOX1 was about ten times higher than for Pv18S rRNA. Infections of very low parasitaemia were detected by Pv-mtCOX1 qPCR but not by Pv18S rRNA qPCR. These results confirm the mitochondrial genome as a suitable target for achieving a substantially more sensitive qPCR assay, enabling detection of scarce mitochondrial template copies in very low density infections.

## Loop-mediated isothermal amplification (LAMP)

LAMP assays amplify single-copy or multi-copy molecular markers in an isothermal reaction. This method seems optimally suited for application at the point-of-care (POC) in field settings. LAMP requires little in the way of equipment and may be carried out by mobile laboratories. LAMP is suitable for detecting sub-microscopic infections [34, 35]. However, LAMP cannot quantify parasitaemia and some protocols for measuring amplification are not very robust, such as hydroxynaphtol blue detection (Additional file 6: Figure S2). The use of fluorescent dyes to detect LAMP products can overcome some of the limitations of conventional LAMP detection.

Unspecific template-independent amplification is a long-standing problem in LAMP that has been addressed by a number of authors [36–38]. Amplification artefacts arise from primer complexes formed by the four to six primers per reaction, two of which are very long primers pre-designed for generating loops. Primer dimers or junk amplification products may be generated in negative controls. False-positive LAMP reactions reportedly occurred at random [34]. This phenomenon leads to loss of confidence in results, as this type of amplification artefact cannot be distinguished from reagent contamination. Moreover, it is difficult to validate true positivity at ultralow template concentrations around the limit of detection. Systematic validation is further complicated by chance effects in template distribution in low densities.

To improve the specificity of LAMP, several assay parameters were optimized, such as decreasing the primer concentration, reducing the incubation time of the LAMP reaction, testing different published primer sets, and optimizing primers (unpublished own results) using commercial (Mast Isoplex Malaria Lamp Kit; Mast Diagnostica) as well as home-made master mixes made up of individually purchased reagents (New England Biolabs). False positive results were primarily obtained with primers targeting the 18S rRNA genes of the genus *Plasmodium* [39]. Using alternative primers that target the mitochondrial genome of the genus *Plasmodium* [35], false-positive results in negative controls were substantially reduced but not eliminated. Amplification of LAMP products can be tracked in real-time using a StepOne thermocycler to detect the fluorescent dye of a commercial master mix. In negative controls, signals from unspecific amplification appeared later in the reaction than signals from the true positive reaction observed when a template was present. However, positive samples with low parasite densities equivalent to 1 parasite/µL could not be distinguished from false-positives (Additional file 7: Figure S3). Duration of incubation was a crucial determinant for false-positive results. Some published protocols incubate LAMP for 60 min, for example [39], whereas LAMP kit manuals allot 40 min. To avoid false-positive results, reaction time should not be extended, even though this might result in a potential loss of sensitivity by missing low density infections. When a commercial LAMP Kit with lyophilized primers (EIKEN CHEMICAL CO., LTD) was used, unspecific amplification was only rarely observed.

## Consequences of false-positive and false-negative test results

To guide malaria control and surveillance, reliable prevalence data is of great importance, particularly in areas with low endemicity or in regions recently declared malaria-free. False-positive test results lead to overestimation of residual malaria transmission and may cause unnecessary concerns. In contrast, a large extent of false-negative results would underestimate the true transmission intensity. However, such underestimation is usually expected, as epidemiologists and public health workers are well aware of imperfect malaria diagnosis.

What should be the guiding principle for selecting the most suitable diagnostic test? The dilemma consists in a trade-off between sensitivity and false-positivity, as seen in selecting the incubation time for a LAMP reaction, or in the use of RNA-based parasite detection by qRT-PCR. Evidently, the conservative and more stringent results are preferable because the detectability of parasites in ultra-low infections is always imperfect. The most appropriate nucleic acid amplification technique (NAAT) must be chosen in consideration of the task in question. For example, for focal screen and treat or surveillance-response activities, high sensitivity might be more important than an occasional false-positive result. Thus, any decision about which diagnostic methods to use should be aligned with each specific task and consider the limitations of the diagnostics applied.

It is important to keep in mind that stochastic variation in results is always observed when infections are around the limit of detection of a given assay. For example, when 150 samples collected in PNG were screened three times with the same *P. vivax* 18S rRNA assay, only 14 infections were detected in all three replicates, while 19 infections were detected in only one or two replicates. This variation was less pronounced for *P. falciparum* (24/31 infections detected in all three replicates), most likely due to the overall higher density of *P. falciparum* [12]. Thus, when results are compared between field laboratories and reference laboratories, slightly different results are expected, even when using the same protocols. Only if a laboratory repeatedly detects fewer infections compared to a reference laboratory do lab-specific procedures have to be optimized.

## Relevance of detecting ultralow parasite densities

The limited resources in malaria endemic areas warrant a discussion on whether molecular diagnostics and the establishment of qPCR assays in field laboratories are needed. For *P. vivax,* NAAT seem more necessary than for *P. falciparum*. The *P. vivax*-specific diagnostic challenges include lower mean parasite densities, less sensitive RDTs and a greater need for diagnosis of all infections to prevent later relapses and, thus, continued transmission [8]. These challenges can be tackled to some extent by molecular diagnostics, but all diagnostic methods, including NAAT, sooner or later reach a test-specific limit of detection. Test sensitivity largely depends on the volume of blood used for DNA or RNA extraction and on the whole blood equivalent added to the amplification reaction. Increasing test sensitivity beyond the current levels of detection would require venous blood samples and white blood cell depletion [40]. That option is not considered feasible for large-scale field surveys. Thus, detection of malaria parasites remains imperfect.

However, if capacity, equipment and reagents were available in *P. vivax* endemic areas, those facilities could act as reference laboratories for quality assurance. This would greatly help to improve diagnostic quality in research and surveillance. The answer to the question about molecular diagnostics being essential or not largely depends on the specific task, be it rapid reactive response or general surveillance, research or clinical trial.

The usage of molecular diagnosis for understanding the reservoir of transmission and to guide interventions has been emphasized by many recent publications [41–45], but the epidemiological relevance of detecting submicroscopic *P. vivax* infections is not the primary focus of this publication.

## Gametocytes in low density *P. vivax* infections

Treatment of asymptomatic *P. vivax* infections has two aims: firstly, to target gametocytes to prevent onward transmission to mosquitoes and, secondly, to target dormant liver stages to prevent relapses. Blood-stage infections originating from relapses frequently carry gametocytes and, thus, likely also contribute to transmission [46].

**Fig. 5:**
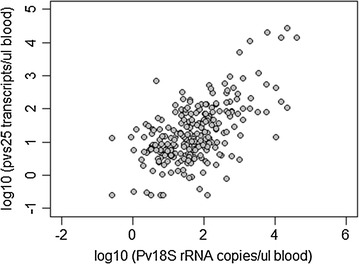
Correlation between *P. vivax* parasite density measured by 18S rRNA qPCR and *P. vivax* gametocyte density determined as *pvs25* transcript numbers by qRT-PCR (graph based on data originally published in [2])

In the context of transmission control, the question arises whether all low intensity *P. vivax* infections carry gametocytes and whether molecular tools are required to determine the prevalence of gametocytes in the population. *Plasmodium vivax* gametocytes are detected either by LM or by quantifying transcripts of genes that are specifically expressed in *P. vivax* gametocytes. *P. vivax* gametocytes are difficult to distinguish from trophozoites by LM. Molecular detection of gametocytes is more sensitive and more precise. The standard marker gene *pvs25* encodes an ookinete surface protein. Quantitative reverse transcription PCR (qRT-PCR) is performed on RNA extracted from a blood sample [10]. Gametocyte detection in field surveys is complicated by the requirement for appropriate RNA-stabilizing procedures, such as immediate transfer of a blood sample into a stabilizing reagent [10]. When *pvs25* transcript numbers were plotted against Pv18S rRNA gene copies, a moderate correlation (R = 0.59) was observed in samples from two cross-sectional community surveys conducted in PNG (Fig. 5) [2, 41]. A much stronger correlation (r^2^ = 0.82) was observed in a study from Thailand, using the same diagnostic methods [44]. *P. vivax* gametocytes can be detected within 3 days of the appearance of asexual parasites in the blood [47]. This also argues for using *P. vivax* blood stage parasites as a surrogate marker for gametocytaemia. Performing gametocyte detection and quantification assays are not required for surveillance.

Asymptomatic infections were often found to carry gametocytes in studies in Brazil, Thailand and PNG [2, 44, 48]. The global trend of declining *P. vivax* malaria entails reduced mean population parasite densities and, thus, a lower proportion of infections carrying detectable gametocytes [2]. How much these low density infections contribute to transmission remains unclear. An indication that low density infections may be relevant derives from mosquito feeding assays conducted in Thailand, showing that *P. vivax* infections < 10 parasites/µL can be infective, though only rarely and yielding few oocyts [49]. Similarly, mosquito feeding assays will be required to determine the infectivity of *P. vivax* gametocytes following drug treatment. Transmission during convalescence might be relevant in elimination settings, where levels of acquired immunity are low and therefore a large proportion of all those infected will seek treatment.

## Conclusions

Key points with particular relevance for *P. vivax* diagnosis in community samples:

- The input blood volume determines test sensitivity. To improve test performance, the volume of finger prick blood processed or DNA and RNA template added to NAAT should always be maximized.
- Multi-copy targets used for qPCR are superior for detection and necessary for pooling samples before molecular analysis. The tenfold increase of PCR templates per cell when using the Pv-mtCOX1 assay led to gains in positivity and more precise prevalence estimates in a cross-sectional survey in Brazil.
- The suitability of RNA-based assays is questionable for processing large-scale field samples with a wide range of parasite densities. A fully enclosed system for sample processing and tight controls seem critical to avoid false-positivity.
- Different numbers of genomes per *P. vivax* blood stage do not permit simple quantification of parasitaemia or gametocytaemia. The most robust quantification consists of the copy numbers of the molecular marker detected per µL of whole blood equivalent.
- There is no need for specific gametocyte assays in surveillance and monitoring of interventions, as *P. vivax* asexual densities and gametocyte densities are well correlated.
- Some limitations for NAAT cannot be resolved, such as imperfect detection derived from restrictions in blood volume, sampling procedures in the field or chance effects in detecting a very low abundant PCR template.
- It is important to investigate methodological limitations and shortfalls of the diagnostic techniques used and consider their effects on clinical trial outcomes, as well as on the planning of interventions.

## Additional files

**Additional file 1: Table S1.** Gene IDs of *P. vivax* 18S rRNA (small subunit rRNA gene).

**Additional file 2: Table S2.** Assay conditions for *P. vivax* qPCR targeting cox1 (Pv-mtCOX1 assay).

**Additional file 3: Table S3.** Performance Pv-mtCOX1 qPCR.

**Additional file 4: Table S4.** Limit of detection of Pv-mtCOX1.

**Additional file 5: Figure S1.** Fold-difference in template copy numbers detected by molecular marker Pv-mtCOX1 versus marker Pv18S rRNA.

**Additional file 6: Figure S2.** LAMP reaction detected with hydroxynaphtol blue (HNB).

**Additional file 7: Figure S3.** Real-time LAMP reaction with calcein detection.

## Abbreviations

cytB: cytochrome B
cox1: cytochrome C oxidase I
cox3: cytochrome C oxidase III
ddPCR: digital droplet PCR
LM: light microscopy
LAMP: loop-mediated isothermal amplification
NAAT: nucleic acid amplification technique
mtCOX1: mitochondrial cytochrome C oxidase 1
mtDNA: mitochondrial DNA
PCR: polymerase chain reaction
PNG: Papua New Guinea
POC: point-of-care
qPCR: quantitative PCR
qRT-PCR: quantitative reverse transcription PCR
RDT: rapid diagnostic test
RFLP: restriction fragment length polymorphism
TPP: target product profile
