# Supplementary material for: Plasmodium vivax molecular diagnostics in community surveys: pitfalls and solutions
Source: Malar J. 2018 Jan 30;17:55. doi: 10.1186/s12936-018-2201-0 (PMC5789620; doi:10.1186/s12936-018-2201-0)
Supplement: Supplementary file 1 — Additional file 1: Table S1. Gene IDs of P. vivax 18S rRNA (small subunit rRNA gene). [file 12936_2018_2201_MOESM1_ESM.docx]

**Additional file 1**

**Table S1: Gene IDs of *Plasmodium vivax* 18S rRNA** (small subunit rRNA gene)

| **Gene ID** | **Strain** | **Predicted function** | **Source** |
| --- | --- | --- | --- |
| **PVP01_0202900*** | **P01** | **Pv_A-type** (blood stages: asexual & gametocyte) | PlasmoDB |
| PVP01_0504700 | P01 | Pv_O-type (oocyst) | PlasmoDB |
| PVP01_0622800 | P01 | Pv_S-type (sporozoite) | PlasmoDB |
| PVP01_0801900 | P01 | No wrongly annotated in PlasmoDB | PlasmoDB |
| **U07367.1*** | Sal1 | **Pv_A-type** (blood stages: asexual & gametocyte) | Li et al. 1997 |
| U93095.1 | Sal1 | Pv_O-type (oocyst) | Li et al. 1997 |
| U07368.1 | Sal1 | Pv_S-type (sporozoite) previous C | Li et al. 1997 |

*Target sequence of assay used in the present study (details published in [1]).

1. Wampfler R, Mwingira F, Javati S, Robinson L, Betuela I, Siba P, Beck HP, Mueller I, Felger I: **Strategies for detection of Plasmodium species gametocytes.** *PLoS One* 2013, **8:**e76316.
