# Supplementary material for: Plasmodium vivax molecular diagnostics in community surveys: pitfalls and solutions
Source: Malar J. 2018 Jan 30;17:55. doi: 10.1186/s12936-018-2201-0 (PMC5789620; doi:10.1186/s12936-018-2201-0)
Supplement: Supplementary file 2 — Additional file 2: Table S2. Assay conditions for P. vivax qPCR targeting cox1 (Pv-mtCOX1 assay). [file 12936_2018_2201_MOESM2_ESM.docx]

**Additional file 2**

Assay conditions for *P. vivax* qPCR targeting *cox1* (Pv-mtCOX1 assay)

Table S2: Pv-mtCOX1 assay set up

|  | Pv-mtCOX1 |
| --- | --- |
| Pv-mtcox1-fw | 5’-TTATATCCACCATTAAGTACATCACTT-3’ |
| Pv-mtcox1-rev | 5’-AACCTTTAGATCTTAGATGCATTACA-3’ |
| Pv-mtcox1-probe | 5'-FAM-CCTGTTGCAGTAGATGTTATCATTG-BHQ1-3’ |
|  |  |
| Total volume | 12 |
| DNA volume | 4 |
| TaqMan^®^ Gene Expression Mastermix (Thermo Fisher Scientific) | 1x |
| Primer (fw & rev) | 800 nM |
| Probe | 400 nM |
|  |  |
| Pre-incubation | 2 min – 50°C |
| Initial denaturation | 10 min – 95°C |
| Denaturation | 15 sec – 95 °C |
| Annealing & Elongation | 1 min – 60°C |
| Number of cycles | 45 |
|  |  |
| Positivity threshold | 0.07 |
| Standard material for quantification | Plasmid |
| Platform | StepOne Plus^®^ Real-Time PCR System (Applied Biosystems) |

Primers and probe do not differentiate between *P. vivax* and a zoonotic *P. vivax* variant or distinct species (*P. simium*) found in the South of Brazil [2].

2. Brasil P, Zalis MG, de Pina-Costa A, Siqueira AM, Junior CB, Silva S, Areas ALL, Pelajo-Machado M, de Alvarenga DAM, da Silva Santelli ACF, et al: **Outbreak of human malaria caused by Plasmodium simium in the Atlantic Forest in Rio de Janeiro: a molecular epidemiological investigation.** *Lancet Glob Health* 2017, **5:**e1038-e1046.
