# Supplementary material for: Plasmodium vivax molecular diagnostics in community surveys: pitfalls and solutions
Source: Malar J. 2018 Jan 30;17:55. doi: 10.1186/s12936-018-2201-0 (PMC5789620; doi:10.1186/s12936-018-2201-0)
Supplement: Supplementary file 3 — Additional file 3: Table S3. Performance Pv-mtCOX1 qPCR. [file 12936_2018_2201_MOESM3_ESM.docx]

**Additional file 3**

**Performance** **Pv-mtCOX1 qPCR**

For standard curves and internal controls a control plasmid containing the *P. vivax* *cox1* amplicon was generated. The *cox1* PCR product was amplified from *P. vivax* genomic DNA and cloned into the TOPO TA vector (Invitrogen). Analytical sensitivity and qPCR efficiency was determined on a plasmid dilution row in the range from 10^6^-0.01 copy numbers/µL. The performance parameters of the Pv-mtCOX1 qPCR in comparison to the earlier published *P. vivax* 18S rRNA assay [1] are shown in **Table S3**.

**Table S3: Performance parameters for *P. vivax* 18S rRNA qPCR and Pv-mtCOX1 qPCR**

| Target gene | Assay | Slope | Efficiency | Intercept | R^2^ | Amplicon size | Amplified copy numbers /  genome | LOD  Plasmid copies/µL DNA solution |
| --- | --- | --- | --- | --- | --- | --- | --- | --- |
| 18S rRNA | TaqMan | -3.52 | 92.25 | 42.39 | 0.99 | 216 bp | 1* | 1 |
| mtCOX1 | TaqMan | -3.32 | 100% | 36.18 | 0.97 | 148 bp | variable** | 0.1 |

*1 out of 3 *P.vivax P01* 18S rRNA sequences

** mtCOX1 copy numbers depend on abundance of late parasite stages in blood sample

1. Wampfler R, Mwingira F, Javati S, Robinson L, Betuela I, Siba P, Beck HP, Mueller I, Felger I: **Strategies for detection of Plasmodium species gametocytes.** *PLoS One* 2013, **8:**e76316.
