# Supplementary material for: Plasmodium vivax molecular diagnostics in community surveys: pitfalls and solutions
Source: Malar J. 2018 Jan 30;17:55. doi: 10.1186/s12936-018-2201-0 (PMC5789620; doi:10.1186/s12936-018-2201-0)
Supplement: Supplementary file 4 — Additional file 4: Table S4. Limit of detection of Pv-mtCOX1. [file 12936_2018_2201_MOESM4_ESM.docx]

**Additional file 4**

**Limit of Detection of Pv-mtCOX1**

**Table S4: Serial dilution of Pv-mtCOX1 plasmid**

| Plasmid | Pv-mtCOX1 | |
| --- | --- | --- |
| Copies/µL DNA solution* | Positivity | Mean C_t_ (±StDev) |
| 1000000 | 3/3 | 17.6±0.2 |
| 100000 | 3/3 | 20.3±0.14 |
| 10000 | 3/3 | 23.8±0.3 |
| 1000 | 5/5 | 26.3±0.3 |
| 100 | 5/5 | 29.5±0.2 |
| 10 | 5/5 | 32.7±0.1 |
| 5 | 4/5 | 33.8±0.8 |
| 2 | 5/5 | 35.2±0.6 |
| 1 | 5/5 | 36.6±0.7 |
| 0.5 | 5/5 | 37.0±1.5 |
| 0.1 | 3/5 | 38.2±1.6 |
| 0.01 | 0/5 | n.a. |

*4µL template DNA were added per PCR reaction
