# Supplementary material for: Plasmodium vivax molecular diagnostics in community surveys: pitfalls and solutions
Source: Malar J. 2018 Jan 30;17:55. doi: 10.1186/s12936-018-2201-0 (PMC5789620; doi:10.1186/s12936-018-2201-0)
Supplement: Supplementary file 5 — Additional file 5: Figure S1. Fold-difference in template copy numbers detected by molecular marker Pv-mtCOX1 versus marker Pv18S rRNA. [file 12936_2018_2201_MOESM5_ESM.docx]

**Additional file 5**

**Figure S1**


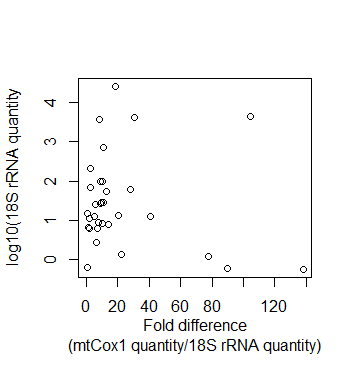


**Figure S1:** Fold-difference^1)^ in template copy numbers detected by molecular marker Pv-mtCOX1 versus marker Pv18S rRNA.

^1)^ Median: 9.7-fold higher copy numbers by Pv-mtCOX1 versus marker Pv18S rRNA.

Mean: 22.5-fold higher copy numbers by Pv-mtCOX1 versus marker Pv18S rRNA.

The mean estimate was influenced by few outliers. In three low-density samples chance effects in template distribution generally plays a critical role
