# Supplementary material for: Plasmodium vivax molecular diagnostics in community surveys: pitfalls and solutions
Source: Malar J. 2018 Jan 30;17:55. doi: 10.1186/s12936-018-2201-0 (PMC5789620; doi:10.1186/s12936-018-2201-0)
Supplement: Supplementary file 6 — Additional file 6: Figure S2. LAMP reaction detected with hydroxynaphtol blue (HNB). [file 12936_2018_2201_MOESM6_ESM.docx]

**Additional file 6**

**A**


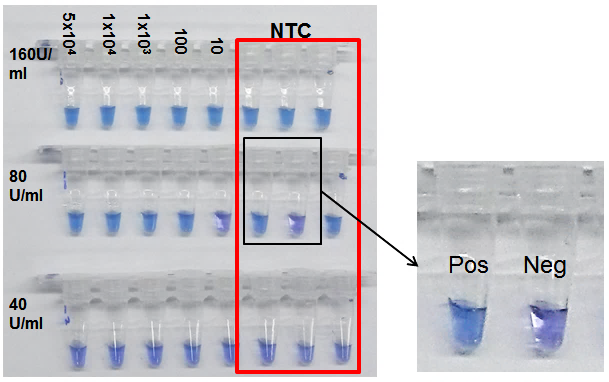


**Figure S2A:** LAMP reaction detected with HNB using primers published by Han et al. 2007 targeting the 18S rRNA gene of the genus Plasmodium, tested in a *P. falciparum* 3D7 ring stage  *in vitro* culture dilution row, ranging from the equivalent of 5x10^4^ to 10 parasites per microliter with different concentrations of Bst 3.0 polymerase.
