# Supplementary material for: Plasmodium vivax molecular diagnostics in community surveys: pitfalls and solutions
Source: Malar J. 2018 Jan 30;17:55. doi: 10.1186/s12936-018-2201-0 (PMC5789620; doi:10.1186/s12936-018-2201-0)
Supplement: Supplementary file 7 — Additional file 7: Figure S3. Real-time LAMP reaction with calcein detection. [file 12936_2018_2201_MOESM7_ESM.docx]

**Additional file 7**


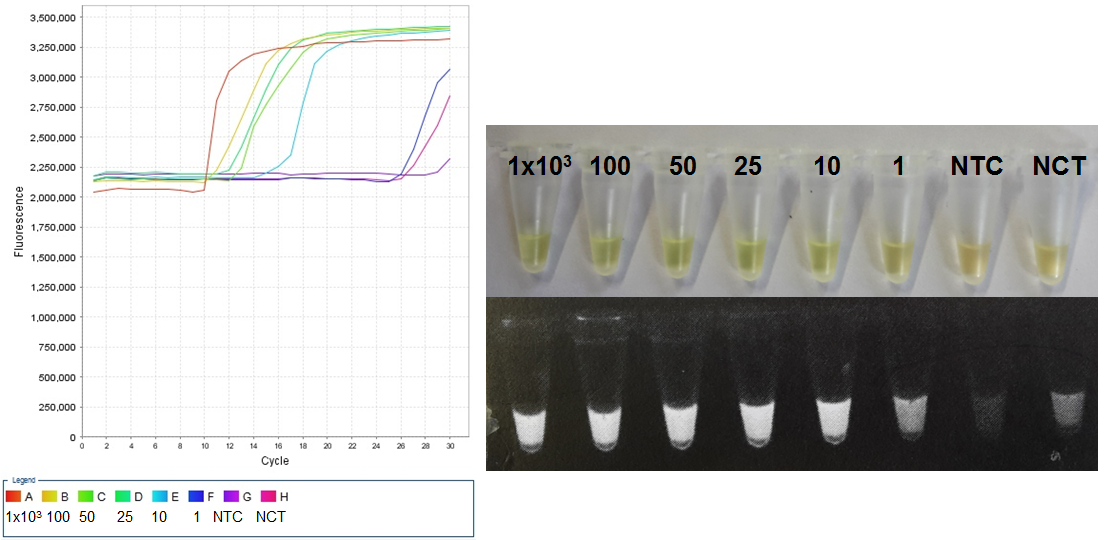


**Figure S3:** Real-time LAMP reaction with Calcein detection using primers of Polley et al. 2010 targeting mitochondrial DNA of the genus Plasmodium, tested in a *P. falciparum* 3D7 ring stage *in vitro* culture dilution row, ranging from the equivalent of 1000 to 1 parasites per microliter with different concentration of Bst 2.0 WarmStart polymerase. a) Fluorescence measured real-time using a StepOne Real-time PCR thermocycler. b) Naked eye visualization. c) Detection in a fluorescent cabin. Colour code for curves: (A) 103, (B) 100, (C) 50, (D) 25, (E) 10, (F) 1, (G & H) No Template Control (NTC).
